# Determining a NOAEL for the consortium linking academic and regulatory insights on BPA toxicity (CLARITY-BPA) core study

**DOI:** 10.3389/ftox.2025.1639737

**Published:** 2025-08-21

**Authors:** Melissa Badding, Noor Aly, Kevin Sondenheimer

**Affiliations:** ^1^ Exponent, Inc., Alexandria, VA, United States; ^2^ Covestro Deutschland AG, Leverkusen, Germany

**Keywords:** bisphenol A (BPA), CLARITY-BPA, no-observed-adverse-effect level (NOAEL), risk assessment, reproductive toxicity

## Abstract

The CLARITY-BPA Core Study is the most comprehensive animal study of oral bisphenol A (BPA) exposure to date. Rats were exposed daily, *in utero* until postnatal day 21 or for the animals’ lifetime. While the study authors concluded that several observations at the highest dose may be BPA treatment-related, a No-Observed-Adverse-Effect Level (NOAEL) has not been proposed in the published reports. Therefore, select endpoints deemed by the study authors to be potentially BPA treatment-related were further evaluated to determine a NOAEL. These include findings in the female reproductive tract and male pituitary gland at the highest dose level (25,000 μg/kg-bw/day). The data were examined for dose-response, relevance, and consistency of findings across study arms and timepoints, histopathological progression, and concordance with the estradiol positive control. Based on our evaluation, some of the female reproductive tract findings are possibly BPA treatment-related. However, there is a lack of consistency between study arms and/or timepoints, no significant dose-response, and a lack of progression to tumors for proliferative findings. Finally, the findings from the Core Study agree with previous high-quality guideline studies which determined that BPA did not pose adverse effects at doses below 25,000 μg/kg-bw/day in rodents. Altogether, some findings from the Core Study may be BPA treatment-related but they should not be considered adverse. Therefore, we hypothesize that the NOAEL from the Core Study is reasonably considered to be 25,000 μg/kg-bw/day.

## 1 Introduction

Bisphenol A (BPA; CASRN 80–05–7) is a high-production volume industrial chemical mainly used as monomer in the production of polycarbonate plastic and epoxy resins. These BPA-based products have broad applications in consumer products, including medical devices and storage containers for foods and beverages. Despite extensive research into BPA toxicity, there remains a significant scientific debate regarding a safe exposure level for humans. Regulatory agencies in North America, Australia and Asia have concluded that current dietary BPA exposure levels of the general population are low (<0.5 μg/kg-bw/day) and do not pose significant risks to human health (United States Food and Drug Administration (FDA), 2014a; United States Food and Drug Administration (FDA), 2014b; Health Canada, 2012; Choi et al., 2010; Park et al., 2016; FSANZ, 2024). However, the European Food Safety Authority (EFSA) recently published a re-evaluation of the risk of BPA in foodstuffs, in which a revised tolerable daily intake (TDI) of 0.2 ng BPA/kg-bw/day was derived and concluded that there is a health concern from dietary BPA exposure (EFSA, 2023).

The Consortium Linking Academic and Regulatory Insights on Bisphenol A Toxicity (CLARITY-BPA) was developed by the U.S. National Toxicology Program (NTP), U.S. National Institute of Environmental Health Science (NIEHS), and U.S. Food and Drug Administration (FDA) in 2010, and the CLARITY-BPA study convened in 2012. The draft Core Study report was first released in 2018, with the final compendium report published in 2021 (NTP, 2018; NTP, 2021). The studies conducted under the CLARITY-BPA program were designed to study the full range of potential health effects from exposure to BPA. The main study (called the “Core Study”) was a GLP (Good Laboratory Practice) and guideline-compliant 2-year in utero through chronic oral exposure toxicity study in rats. There were also fourteen NIEHS-funded university-based researchers (called the “Grantees”) that conducted supporting investigational studies with endpoints using tissues and animals raised under same the condition as the animals in the Core Study.

**FIGURE 1:**
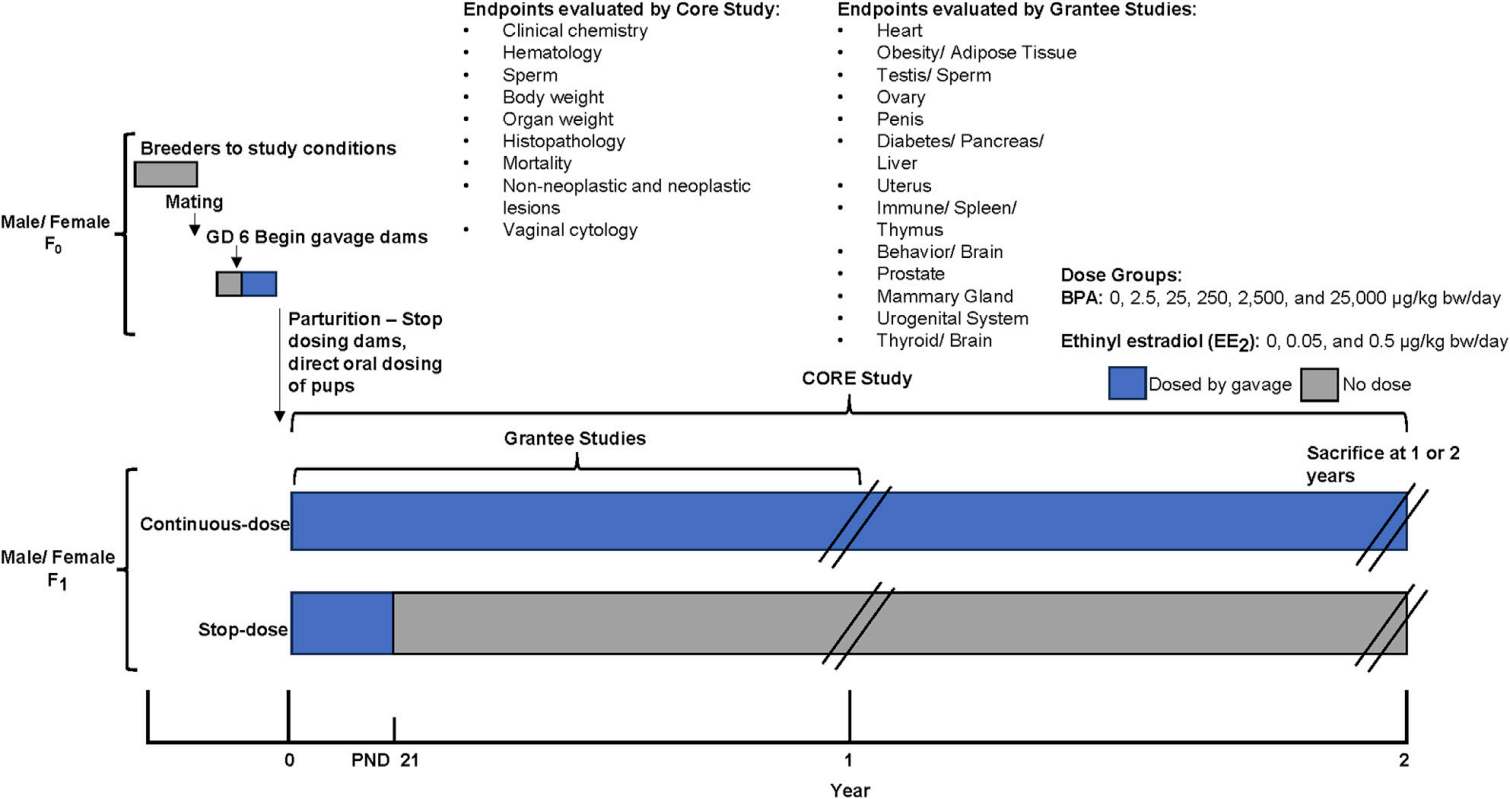
CLARITY-BPA Core Study design, modified from NTP (2021). The Core Study had two exposure duration arms: a stop-dose arm (treatment from gestation through weaning - PND 21, ending at 1 or 2 years) and a continuous-dose arm (treatment from gestation through lifetime, ending at 1 or 2 years). Pregnant Sprague-Dawley rats received BPA by oral gavage from gestation day 6 until labor, with pups dosed from postnatal day 1. The study included five BPA dose groups (2.5–25,000 µg/kg-bw/day), two ethinyl estradiol (EE_2_) groups (0.05 and 0.5 µg/kg-bw/day) as positive controls, and a vehicle control group. The Core Study evaluated endpoints including clinical chemistry, hematology, sperm parameters, body and organ weights, histopathology, mortality, neoplastic and non-neoplastic lesions, and vaginal cytology. Additionally, Grantee studies evaluated particular endpoints in animals raised under the same conditions as the Core Study.

The Core Study was designed with two exposure duration arms: a stop-dose arm (gestation through weaning) and a continuous-dose arm (gestation through sacrifice), which both ended at 1 or 2 years. This design allows a comparison of the treatment effects following gestational through perinatal exposure with those of gestational through lifetime exposure, thus partially addressing the impact of early exposure alone relative to a lifetime of continuous exposure. In both study arms, BPA was administered by oral gavage to pregnant Sprague-Dawley rats (from the NCTR breeding colony: Sprague-Dawley/CD23/NctrBR) from gestation day (GD) 6 through the beginning of labor (see Figure 1). Pups were directly dosed by oral gavage beginning on postnatal (PND) day 1. Thereafter, the dosing differed for the two study arms, with each arm having animals sacrificed at one and 2 years. The continuous-dose arm included BPA exposure until sacrifice while the stop-dose arm had treatment ceasing at PND 21. The study included five BPA treatment groups with doses ranging from 2.5 to 25,000 µg/kg-bw/day, two ethinyl estradiol (EE_2_) treatment groups with doses of 0.05 and 0.5 µg/kg-bw/day, and a vehicle control (0.3% carboxymethylcellulose) group. The EE_2_ groups were included as a positive control to compare for low-dose estrogenic effects. For further information on the Core Study design with an overview on the investigated endpoints, dose selection and data collection please refer to Schug et al. (2013) and Heindel et al. (2015).

The authors of the final CLARITY-BPA Core Study report stated that, “In conclusion, in the CLARITY-BPA core study, statistical differences between BPA treatment groups, particularly below 25,000 μg/kg-bw/day, and the vehicle control group detected by the low-stringency statistical tests applied to histopathology lesions, were not dose responsive, sometimes occurring in only one low or intermediate dose group, and did not demonstrate a clear pattern of consistent responses within or across organs within the stop- and continuous-dose arms and sacrifice times. In contrast, the high EE_2_-dose elicited several estrogenic effects in females in a clearly interpretable and biologically plausible manner. Several observations at 25,000 μg BPA/kg-bw/day may be treatment-related, including effects mentioned above in the female reproductive tract (ovary, uterus, and vagina) and in the male pituitary.” (NTP,2018).

Similarly, the authors of the subsequently published manuscript by Camacho et al. (2019) concluded that, “BPA did not elicit adverse effects in the in-life or terminal endpoints monitored in either sex below 25,000 μg/kg bw/day.” and “The lack of consistent responses within the continuous- and stop-dose arms within and across tissues brought into question the plausible relationship of most of these lesions to BPA treatment. There was a possible relationship between the increased incidences of lesions in the female reproductive tract and the male pituitary and exposure to the 25,000 μg BPA/kg-bw/day dose level.”

The Core Study authors did not discuss which of the endpoints are considered to be related to BPA treatment, only that the relationship is “possible” or “may be treatment-related” (Camacho et al., 2019; NTP,2018). Additionally, the study authors did not evaluate the adversity and toxicological relevance of these findings. Furthermore, neither publication of the CLARITY-BPA Core Study results proposed a No-Observed-Adverse-Effect Level (NOAEL) for this study. We aim to determine a scientifically appropriate NOAEL for the CLARITY-BPA Core Study by further evaluating the relationship to BPA treatment, and toxicological relevance for the statistically significant findings in the ovary, uterus, vagina, and pituitary gland identified by the Core Study authors.

## 2 Endpoints included in the evaluation

The findings included in the present evaluation are those described by the Core Study authors as being statistically significant from vehicle control groups at the highest BPA dose level in primary tests and that were potentially related to BPA treatment (see quote in NTP,2018: “Several observations at 25,000 μg BPA/kg-bw/day may be treatment-related, including effects mentioned above in the female reproductive tract (ovary, uterus, and vagina) and in the male pituitary.”). In addition to the text in the published CLARITY-BPA Core Study report, Table 1 (Summary of Endpoints Evaluated and Statistically Significant Treatment Effects of BPA and EE_2_ Relative to Vehicle Controls) was reviewed and consulted for this determination. The full statistical analysis reports can be found in Supplemental Appendices XVII-XXXI, XXXIII, and XXXIV of the CLARITY-BPA Core Study report (NTP,2018). In short, statistical tests were conducted at the 0.05 significance level and, in most cases, were two-sided. Trend tests for treatment effect (either increased or decreased relative to vehicle control) with increasing dose were conducted only for vehicle control and BPA treatment groups.

**TABLE 1:** Mean absolute ovary weight values at interim sacrifice in stop and continuous dosing arms.

	Absolute ovary weight (mean) (mg)			
BPA dose (µg/kg-bw/day)	Stop-dose	Number of animals	Continuous-dose	Number of animals
(Vehicle control)	157	20	140	20
2.5	149	22	147	22
25	148	20	142	17
250	147	22	138	21
2,500	147	20	140	18
25,000	136^a^	20	140	21

^a^ Significant dose trends for the absolute ovarian weight were reported compared with the vehicle control.

Therefore, the following endpoints at the 25,000 μg BPA/kg-bw/day dose level, in the female reproductive tract and in the male pituitary gland were included:

• Ovary - decreased absolute weight and ratio to brain weight in females of the stop-dose study arm at the 1-year timepoint.
  - follicle cysts in females of the stop-dose study arm at the 1-year timepoint.
• Uterus - epithelial cell apoptosis in females of the continuous-dose study arm at the 1-year timepoint.
• Vagina - epithelial hyperplasia in females of the continuous-dose study arm at both the 1-year and 2-year timepoints.
• Pituitary gland - pars distalis hyperplasia in males of the stop-dose and continuous-dose study arms at the 2-year timepoint.

### 2.1 Evaluation of relationship to BPA treatment

The histopathological observations in the ovary, uterus, vagina and pituitary gland (males only) were first evaluated for whether they are likely to be BPA treatment-related. This was based on an assessment of the consistency of the findings within the Core Study across dosing arms (stop-dose and continuous-dose) and consideration of the appearance at time points (1-year and 2-year sacrifices) and any apparent dose-response. Additionally, evidence of the same endpoints from the CLARITY-BPA Grantee studies (summarized in NTP, 2021) and the 90-day range-finding study from the same group involved in the Core Study (Delclos et al., 2014) was considered, as these studies were performed under the same or similar experimental conditions. The findings were also evaluated for whether they were observed in the EE_2_ treatment groups, in order to determine whether they may be associated with low-dose estrogenic effects. The summary of this evaluation can be found in Table 1.

### 2.2 Evaluation of toxicological relevance

After the evaluation of whether the findings described in Section 2.1 are possibly related to BPA treatment, an evaluation of adversity and/or toxicological relevance was conducted (see Table 2 for summary). This evaluation was based on scientific literature for the specific histopathological findings, progression of any proliferative findings to tumors, and guidelines for reproductive toxicity risk assessment (EPA, 1996; WHO, 2001; OECD, 2008).

**TABLE 2:** Statistically significant high-dose group findings from CLARITY-BPA Core Study and evaluation of whether they are likely BPA treatment-related.

Statistically significant endpoint^a^	Study arm	Time point	BPA dose (µg/kg-bw/day)	Dose-response	Observed in similar studies?	Consistent and related to BPA treatment?
Ovarian absolute weight and ratio to brain weight decrease	Stop-dose	1-year	25,000 + Trend	Not likely^b^	Yes; Delclos et al. (2014) at 300,000 μg/kg/day BPA at PND 21 and 90	Unlikely; not observed in continuous-dose or 2-year BPA groups; however, was observed with EE_2_ treatment and in Delclos et al. (2014) at highest BPA dose
Ovarian follicle cysts	Stop-dose	1-year	25,000 + Trend	Yes	Yes; Delclos et al. (2014) at 300,000 μg/kg/day BPA at PND 90	Possibly; not observed in continuous-dose or 2-year BPA groups, but there appears to be a dose-response and was observed with EE_2_ treatment and in Delclos et al. (2014) at highest BPA dose
Uterine epithelial cell apoptosis	Continuous -dose	1-year	25,000 + Trend	Yes	No	No; not observed in 2-year BPA groups, severity profile reduced at mid-to-high BPA doses compared to control, not observed in Grantee study (Leung et al., 2020) at PND 90 and 1-year time points or in Delclos et al. (2014); however, there appears to be a dose-response and was observed with EE_2_ treatment
Vaginal epithelial hyperplasia	Continuous -dose	1-year	25,000 + Trend	Yes	No	Possibly; not observed in Delclos et al. (2014), but there appears to be a dose-response; also observed at 2-year time point, and was observed with EE_2_ treatment
	Continuous -dose	2-year	25, 2,500 and 25,000 + Trend	No	No	No; severity profile not increased across BPA doses vs control, no dose-response (not SS at 250 μg/kg/day), not observed with EE_2_ at 2 years, and was not observed in Delclos et al. (2014)
Pituitary gland pars distalis hyperplasia	Stop-dose (males)	2-year	25,000 + Trend	No	No	Unclear; not observed in females, no dose-response in either dosing arm, severity profile in stop-dose vehicle control is higher than all BPA groups, and was not observed in Delclos et al. (2014); however, was observed in both treatment arms and in males with EE_2_ treatment
	Continuous -dose (males)	2-year	25,000 + Trend	No	No	

^a^ The full statistical analysis reports can be found in Supplemental Appendices XVII-XXXI, XXXIII, and XXXIV, of the CLARITY-BPA, Core Study report. In short, statistical tests were conducted at the 0.05 significance level and, in most cases, were two-sided. Trend tests for treatment effect (either increased or decreased relative to vehicle control) with increasing dose were conducted only for vehicle control and BPA, treatment groups.

+ Trend: Indicates a significant dose response trend reported.

^b^ The absolute organ weight values for BPA, doses 2.5–2,500 are similar (1.5% difference) (see Table 1), which is not indicative of a trend towards organ weight reduction.

## 3 Evaluation of endpoints

### 3.1 Ovary weight changes and follicle cysts

#### 3.1.1 BPA treatment-related evaluation

In stop-dose females at the 1-year timepoint, significant dose trends for the absolute ovarian weight and ovary weight adjusted for brain weight were reported, and the 25,000 μg BPA/kg-bw/day dose group was significantly lower than the vehicle control weight in both cases. Ovarian weights adjusted for body weight were not statistically different from controls. Additionally, there was a statistically significant increase in ovarian follicle cysts with BPA treatment at 25,000 µg/kg-bw/day together with a significant dose trend at the 1-year timepoint and in the stop-dose arm.

These ovarian findings were only present at the 1-year sacrifice and not at the 2-year sacrifice, and importantly, they were not observed in the continuous-dose arm animals that received BPA treatment throughout their lifetime. Therefore, these findings are evaluated as inconsistent considering exposure duration and the lack of persistence between the earlier and later timepoints.

Moreover, when comparing the absolute ovary weight values in each BPA dosing group, it is obvious that the statistically significant trend (p < 0.05) in ovary weight in the stop-dose group may have only been reached due to a high physiological variance among these animals. Indeed, the measured mean ovary weight in the 25,000 µg/kg-bw/day stop-dose group (136 mg) is about 15% lower than the respective vehicle control value (157 mg); however, when compared to continuous-dose vehicle controls (140 mg), only a 3% difference in ovary weight exists and no statistically significant difference was identified (see Table 3 for comparison).

**TABLE 3:** Findings that may be BPA treatment-related and evaluation of their adversity.

Endpoint	BPA treatment group	Adverse and/or toxicologically relevant?
Ovarian absolute weight and ratio to brain weight decrease	Stop-dose, 1-year	No; ovarian weight changes appear to be reversible since they were not observed at 2-year time point in BPA groups and were not observed with longer (continuous-dose) BPA treatment; also, high variability among the vehicle control groups influenced the statistical analysis
Ovarian follicle cysts	Stop-dose, 1-year	No; common benign lesion in aging rats, appears to be reversible since it was not increased at 2-year time point in BPA groups, and was not observed with longer (continuous-dose) BPA treatment
Vaginal epithelial hyperplasia	Continuous-dose, 1- and 2-year	No; nonspecific change which may occur due to persistent estrus during reproductive senescence and secondary to increases in endogenous estradiol production; however, no change in organ weight, no progression to tumors, and not considered an adverse lesion in the vagina per guidelines
Pituitary gland pars distalis hyperplasia	Stop-dose and continuous-dose (males), 2-year	No; common benign lesion in aging rats, no change in organ weight, no progression to tumors, and not considered adverse lesion in the pituitary gland per guidelines; also, no downstream sex hormonal effects (e.g., sperm parameters) in either dosing arm

These points all indicate that the ovarian findings in the high BPA dose group females are not likely to be BPA treatment-related in the Core Study.

However, there appears to be a dose-response, indicated by the significant trend analyses reported in the Core Study for the ovarian findings mentioned in Section 3.1.1. In stop-dose animals. Concerning ovary weight, it should be noted that the mean values for the first four BPA dose groups (2.5–2,500 µg/kg-bw/day) only slightly differ (1.5% difference), which is not indicative of a trend towards organ weight reduction. As already mentioned above, the high value for the stop-dose vehicle control group has most likely driven the statistical significance here as well. Nevertheless, reduced ovary weights and increased follicle cysts were also observed to be statistically increased with continuous treatment of EE_2_ at the 0.5 µg/kg-bw/day dose, indicating that this finding could be a result of weak estrogenic activity, and thus, related to BPA treatment. Additionally, reduced ovary weights (absolute and adjusted for brain weight) as well as ovarian follicle cysts were observed in the 90-day Delclos et al. (2014) study at the highest BPA dose of 300,000 µg/kg-bw/day at postnatal day (PND) 90. This dose is, of course, much higher than the 25,000 µg/kg-bw/day dose in the Core Study associated with this finding, and it was not reported in the 90-day study at the lower dose levels.

Therefore, it cannot be ruled out that the incidence of ovarian follicle cysts could be affected by BPA’s low estrogenic activity. On the other hand, the observed organ weight alterations (absolute and ratio to brain weight) are unlikely to be the consequence of BPA exposure as all statistical outcomes may have been influenced by the high mean value in the stop-dose vehicle control.

#### 3.1.2 Adversity and toxicological relevance evaluation

The U.S. Environmental Protection Agency (EPA), the World Health Organisation (WHO), and the Organisation for Economic Co-operation and Development (OECD) have respective guidelines for reproductive toxicity risk assessment regarding the adversity of certain male and female reproductive toxicity effects. According to EPA’s and OECD’s reproductive toxicity guidelines, effects on the ovary that may be considered adverse include changes in organ weight and cystic follicles (EPA, 1996; OECD, 2008). EPA’s guidelines state that the weights of reproductive organs are poorly correlated to body weight except for extreme cases; therefore, reduced absolute weights may be considered adverse.

Cysts of the ovarian follicles arise from secondary follicles that fail to ovulate and are common benign lesions that increase in aging rats (NTP, 2023c). These cysts may be associated with persistent estrus or cystic endometrial hyperplasia (NTP, 2023c). While not statistically significant in primary tests, the 25,000 µg/kg-bw/day BPA stop-dose females at a 16-week timepoint were observed to have an increased number of estrus cycle days (4.42 ± 0.15 days vs 4.08 ± 0.12 days in vehicle control) and at the 1-year timepoint there was an increased incidence of cystic endometrial hyperplasia (32% vs 10% in vehicle control) compared with vehicle controls.

As discussed above in Section 3.1.1., neither increased incidence of ovarian follicular cysts nor decreased absolute ovarian weight were observed in BPA-treated animals at the terminal sacrifice (2-year) or in the continuous-dose group that experienced a much longer exposure to BPA. Reversibility of an effect is an important consideration in the risk assessment for potential reproductive effects (EPA, 1996). Given that these findings were only present at the 1-year sacrifice and not at the 2-year sacrifice, the reversibility must be taken into account. It is unclear whether or how both findings resolved by the 2-year timepoint, and why, with longer BPA treatment in the continuous-dosed animals, the findings were not observed. Furthermore, there is no generally accepted standard of how much of a change in follicle counts should be considered adverse, and evidence of a dose-response trend and a statistically significant change in follicle number only does not indicate a toxicologically relevant effect in humans (OECD, 2008).

Altogether, the available evidence indicates that the observed alterations in ovarian follicle cysts and absolute ovarian weight are not likely to be adverse effects of BPA treatment in the Core Study.

### 3.2 Uterine epithelial cell apoptosis

#### 3.2.1 BPA treatment-related evaluation

A statistically significant increase in epithelial cell apoptosis of the uterus was observed in females treated with BPA at 25,000 µg/kg-bw/day from the continuous-dose study arm at the 1-year timepoint. However, this finding was not observed at the 2-year sacrifice. Although there appears to be a dose-response with BPA treatment and uterine epithelial cell apoptosis, and this finding was observed with EE_2_ treatment, the reduced severity profile at mid-to-high BPA doses compared to controls suggests these effects may not be in fact BPA treatment-related. Additionally, this was not observed in the Grantee study that evaluated potential uterine effects at PND 90 and a 1-year timepoint (Leung et al., 2020) or in the Delclos et al. (2014) study at the PND 90 timepoint.

Altogether, it is unlikely that increased uterine epithelial cell apoptosis is due to BPA treatment in the Core Study. Despite this, a further examination of the adversity and toxicological relevance was conducted.

#### 3.2.2 Adversity and toxicological relevance evaluation

The uterine endometrium is sensitive to the influences of estrogenic substances and extended treatment may lead to hypertrophy and hyperplasia. Effects on the uterus may be considered adverse if there are changes in organ weight, infantile or malformed uterus or cervix, endometrial hyperplasia, hypoplasia, or aplasia, and decreased number of implantation sites (EPA, 1996; WHO, 2001; OECD, 2008). However, none of these were observed at any timepoint at the highest BPA dose group. Apoptosis of the endometrial luminal epithelial cells is a common physiological finding in the cycling uterus (Dixon et al., 2014), and it is not considered to be an adverse effect *per se*.

Taken together, there is no scientific basis to believe that the observed increase in uterine epithelial cell apoptosis should be seen as an adverse effect of BPA treatment in the Core Study.

### 3.3 Vaginal epithelial hyperplasia

#### 3.3.1 BPA treatment-related evaluation

A statistically significant increase in vaginal epithelial hyperplasia was observed in females treated with BPA at 25,000 µg/kg-bw/day in the continuous dose study arm at both the 1-year and 2-year timepoints and at 25 and 2,500 µg/kg-bw/day at only the 2-year time point.

At the 1-year time point in the continuous-dose arm, vaginal epithelial hyperplasia may have been related to BPA treatment. This is because there appeared to be a statistically significant trend with BPA treatment, and the finding was also observed with EE_2_ treatment indicating a potential estrogenic effect. However, the dose-response trend may be a result of the non-statistically significant increase in the number of animals in estrus at the 25,000 µg/kg-bw/day BPA dose in continuously dosed females (31.6% vs 28.5% in vehicle control) at 16 weeks, which could have impacted vaginal and uterine histopathological examinations at the 1-year timepoint. Moreover, it is important to note that vaginal epithelial hyperplasia was not observed in the 90-day study by Delclos et al. (2014).

At the 2-year timepoint, the observed effects were unlikely to be related to BPA treatment. This is due to the severity profile not increasing across BPA doses compared to control. Also, the effects were not statistically significant at the mid-dose level 250 µg/kg-bw/day and there was no dose-response at this timepoint. Additionally, it was not observed with EE_2_ treatment at this timepoint.

Altogether, it is unclear whether increased vaginal epithelial hyperplasia is due to BPA treatment in the Core Study. Specifically, the finding appears to be more likely to be treatment-related at the 1-year timepoint than at the 2-year timepoint. Therefore, a further examination of the adversity and toxicological relevance was conducted.

#### 3.3.2 Adversity and toxicological relevance evaluation

The cyclic nature of the estrous cycle causes normal variations in the vaginal epithelium. If the epithelial thickening is a physiological change associated with the estrous cycle, it should not be diagnosed as hyperplasia according to the NTP Nonneoplastic Lesion Atlas (NTP, 2023b). As stated in Section 3.3.1. Above, the non-statistically significant increase in the number of high-dose animals in estrus could have reasonably affected the vaginal histopathological examinations, at least at interim sacrifice.

It should be noted that variations in the thickness of the vaginal epithelium are nonspecific changes that may occur due to persistent estrus during reproductive senescence and secondary to increases in endogenous estradiol production (NTP, 2023b). The EPA, WHO, and OECD reproductive toxicity guidelines state that effects on the vagina may be considered adverse if there are changes in organ weight, infantile or malformed vagina, hypoplasia or aplasia (incomplete development), altered timing of vaginal opening, and abnormal vaginal smear cytology pattern (EPA, 1996; WHO, 2001; OECD, 2008). None of these effects were observed in the BPA treatment groups, and while the EPA guidelines list hyperplasia as a potential “histologic alteration” that may be observed in the vagina, it is not considered an adverse finding in this tissue. Further, no progression from hyperplasia to tumors of the vagina was reported in the Core Study.

Altogether, the observed increase in vaginal epithelial hyperplasia is not considered to be an adverse effect of BPA treatment in the Core Study.

### 3.4 Pituitary gland pars distalis hyperplasia

#### 3.4.1 BPA treatment-related evaluation

A statistically significant increase in hyperplasia in the pars distalis of the pituitary gland was observed in males treated with BPA at 25,000 µg/kg-bw/day from the continuous-dose and stop-dose study arms at the 2-year sacrifice. It is unclear whether these observations are related to BPA exposure or not. For one, pars distalis hyperplasia was not seen in female rats, and it was not observed at the 1-year timepoint or at any dose in the 90-day Delclos et al. (2014) study, which included higher doses of 100,000 and 300,000 µg BPA/kg-bw/day. Additionally, no clear dose-response was observed in the two dosing arms, and the severity profile in the stop-dose vehicle control was higher than all BPA groups. Nevertheless, this finding was observed in both treatment arms at the high dose (25,000 µg/kg-bw/day) and in males receiving continuous dosing of the positive control EE_2_ treatment.

Altogether, it is unclear whether hyperplasia in the pars distalis of the pituitary gland are due to BPA treatment in the Core Study; however, given the possibility, a further examination of the adversity and toxicological relevance was conducted.

#### 3.4.2 Adversity and toxicological relevance evaluation

Hyperplastic lesions in the pars distalis are commonly seen in rats and increase with age (Brändli-Baiocco et al., 2018; NTP, 2023a). According to the EPA, WHO and OECD reproductive toxicity guidelines, alterations in pituitary gland weight and significant histopathologic damage in the pituitary should be considered as adverse (EPA, 1996; WHO, 2001; OECD, 2008). However, the hyperplasia observed should not be characterized as “damage” since it is a proliferative finding as opposed to other destructive-type findings such as necrosis and apoptosis. While this proliferative lesion may precede tumors, pituitary adenomas were not increased in any of the BPA treatment groups in the Core Study.

Additionally, there were no changes in organ weight, and there were no downstream male sex hormonal effects (e.g., sperm parameters) that could be related to the pituitary hyperplasia finding in either dosing arm. Therefore, the increased incidence of hyperplasia in the pars distalis in male rats should not be considered an adverse effect.

Therefore, the totality of evidence suggests that the observed increase in hyperplasia in the pars distalis of the pituitary gland in males is not an adverse effect of BPA treatment in the Core Study.

## 4 Discussion

BPA is one of the most extensively tested chemicals and a huge database on metabolism, human exposure, potential health effects and environmental aspects exists. Divergent findings and interpretations from BPA toxicological studies have introduced a scientific debate on a safe exposure-level and relevant health effects. To resolve these uncertainties and to better inform regulatory decision-making, the CLARITY-BPA program was initiated. One key component of this program is the CLARITY-BPA Core Study. However, since the publication of the results (NTP,2018; Camacho et al., 2019), a NOAEL for this study has not been proposed in the official reports.

While the Core Study authors concluded that “the core study data do not suggest a plausible hazard of BPA exposure in the lower end of the dose range tested”, on the other hand, “several observations at 25,000 μg BPA/kg-bw/day may be treatment-related” (Camacho et al., 2019; NTP,2018). Therefore, the analysis presented herein focused on evaluating all statistically significant findings (indicated by a primary test) at the highest dose of 25,000 μg/kg-bw/day in female reproductive organs and in male pituitary glands, with a focus on adversity and likelihood to be BPA treatment-related.

Several non-neoplastic findings on the female reproductive system and the male pituitary were found to be statistically significant at the 25,000 µg/kg-bw/day BPA dose. This encompasses lower ovarian weights (absolute and adjusted for brain weight), an increased incidence for ovarian follicle cysts, an increase in apoptosis in uterine tissue, epithelial hyperplasia in the vagina, and hyperplasia in the pars distalis of the male pituitary. For some effects, including the ovarian findings, vaginal epithelial hyperplasia, and pituitary gland pars distalis hyperplasia, it cannot be ruled out that they might be the consequence of BPA treatment. However, there is no evidence that any of these observations are likely to be toxicologically relevant or adverse based on the present evaluation.

Mostly, there was a lack of consistency in findings for the BPA treatment groups as some were observed at the interim timepoint (1 year), but not at the terminal timepoint (2 years), while others were observed in stop-dose animals but not in the continuous-dose treatment arm. Even if it is assumed that such findings are induced by BPA exposure, how could one explain that they did not occur with a longer duration of BPA exposure or that they did not persist beyond 1 year if they are in fact adverse effects of BPA exposure? Although, some of the evaluated effects also occurred in the FDA-conducted 90-day study (Delclos et al., 2014) at much higher doses (300,000 μg/kg-bw/day), none of these findings were reproduced in any of the CLARITY-BPA Grantee studies using animals raised and treated under the same conditions as in the Core Study. It should further be emphasized that most of the effects included in this discussion are proliferative (hyperplasia) lesions that are not neoplastic, and within the Core Study, there was no progression to tumors in these tissues.

The majority of the findings can easily be explained by physiological alterations associated with aging in rats. This was also acknowledged by the NTP as they stated that “*In the histopathological evaluations, there were many non-neoplastic lesions associated with aging in this strain of rats in both males and females* […]” (NTP ,2018). Taken together, our analysis of statistically significant findings at the highest BPA dose level suggests that none should be considered adverse effects. Consequently, the NOAEL for the CLARITY-BPA Core Study should be set at 25,000 µg/kg-bw/day.

Other researchers have reviewed the findings from the CLARITY-BPA program and evaluated at least certain datasets. As an outcome of a plenary session, some CLARITY-BPA Grantee investigators published a mini review on the results of three Grantee studies. The authors claimed to have identified effects across multiple organs at the lower BPA dose range. They listed a variety of statistically significant observations across the three analyzed Grantee studies and the Core Study and proposed a lowest-observed-adverse-effect level (LOAEL) based on the adverse effects in the brain, heart, and ovary as well as increased carcinoma risk in the mammary glands and prostate at the lowest tested dose of 2.5 µg/kg-bw/day (Prins et al., 2019). It should be noted that the findings in the ovaries reported in that review (decreased number of primary follicles and number of total healthy follicles) at 2.5 µg/kg-bw/day were not disrupted by EE_2_, and in fact, the lower dose of EE_2_ (0.05 μg/kg-bw/day) did not affect any ovarian endpoints at any age. In a second publication, several Grantee investigators worked together on an analysis and interpretation from eight out of the 17 published Grantee studies. They came to a similar conclusion as Prins et al. (2019) that BPA showed adverse effects at the lowest dose in the brain, prostate gland, urinary tract, ovary (reduced follicle numbers at PND 21), mammary gland, and heart; therefore, a LOAEL of 2.5 µg/kg-bw/day would be justified (Heindel et al., 2020). The only overlap between the affected organs in the CLARITY-BPA Core Study and those reported by Prins et al. (2019) and Heindel et al. (2020) is the ovary; however, the endpoints showing statistical significance were different (e.g., lower ovarian weights and increased incidence of follicle cysts in the CLARITY-BPA Core Study versus reduced number of ovarian follicles in the publications evaluating the Grantee studies). Therefore, the findings do not align between the CLARITY-BPA Core Study and the Grantee studies regarding ovarian effects.

Another review focused on the relevance of the Core Study findings for Canadians and the need for an updated BPA risk assessment. The author discussed exclusively the effects at the low BPA doses as being potentially relevant and pointed on the need for a revision of the current safety threshold (Rogers, 2021).

In each of these publications, the authors raised serious concerns based on the number of statistically significant effects, particularly at low BPA doses, that have been found in the Core Study and the Grantee studies. However, what was not considered in these analyses is the fact that several hundreds of endpoints were measured in the Core Study, and that for this immense number of measurements, remarkably few statistically significant alterations were detected. Moreover, it should be clear that the mere existence of a number of positive statistical outcomes across a study does not constitute proof of a biologically relevant effect. Such a mathematical result needs first to be toxicologically interpreted before any conclusion can be drawn.

A key argument in the discussions of these academic publications on the CLARITY-BPA studies is the proposed existence of a non-monotonic dose response (NMDR) for BPA. As many findings at lower doses were not observed at the higher doses, NMDRs could provide an explanation for this unclear pattern. However, it is important to note that in the Core Study, statistical tests that do not assume monotonicity were used to test for NMDR effects. Importantly, the Core Study authors stated that *“[…] the Core Study data do not suggest a plausible hazard of BPA exposure in the lower end of the dose range”* (Camacho et al., 2019). In addition, a comprehensive analysis for potential non-monotonicity was conducted based on the results of the Core Study. By using the established and systematic modeling approaches of Beausoleil et al. (2016) and Varret et al. (2018), all statistically significant findings (based on primary statistical tests) from the Core Study were evaluated, and the authors of the published study concluded that an NMDR for BPA is unlikely to have been present in the Core Study (Badding et al., 2019). Taken together, the conclusion of Prins et al. (2019), Heindel et al. (2020), and Rogers (2021) that BPA produced potential NMDR effects in the Core Study are in clear contradiction to the interpretation of the Core Study authors (Camacho et al., 2019) and others (Badding et al., 2019).

Since the Core Study report was published, EFSA re-evaluated the risks of BPA in food contact materials (EFSA, 2023). For this assessment, all available data from the CLARITY-BPA program were evaluated. Several observations from the Core Study were forwarded for reference point analysis as potential starting points for the TDI derivation. Beside the herein analyzed observations, one additional finding from the 25,000 µg BPA/kg-bw/day dose groups was proposed to be of relevance: an increased number of exfoliated germ cells in the epididymis in continuously-dosed interim sacrificed (1-year) animals. This endpoint was not included in the present evaluation, since it was not mentioned by the Core Study authors in their conclusion statement about which effects may be BPA treatment-related (e.g., female reproductive organs and male pituitary). Also, the Core Study report indicates no dose-response for this finding (see Table 88 in NTP,2018), as it was only observed at the 1-year timepoint (did not persist out to 2 years with continuous BPA dosing). The authors state that, “For the epididymal lesions, there were no potentially related lesions noted in the testes to increase confidence in this observation as an effect of toxicological significance” (NTP,2018). Therefore, it was not deemed necessary to include this finding for a full evaluation of its likelihood to be BPA treatment-related and its potential adversity.

**FIGURE 2:**
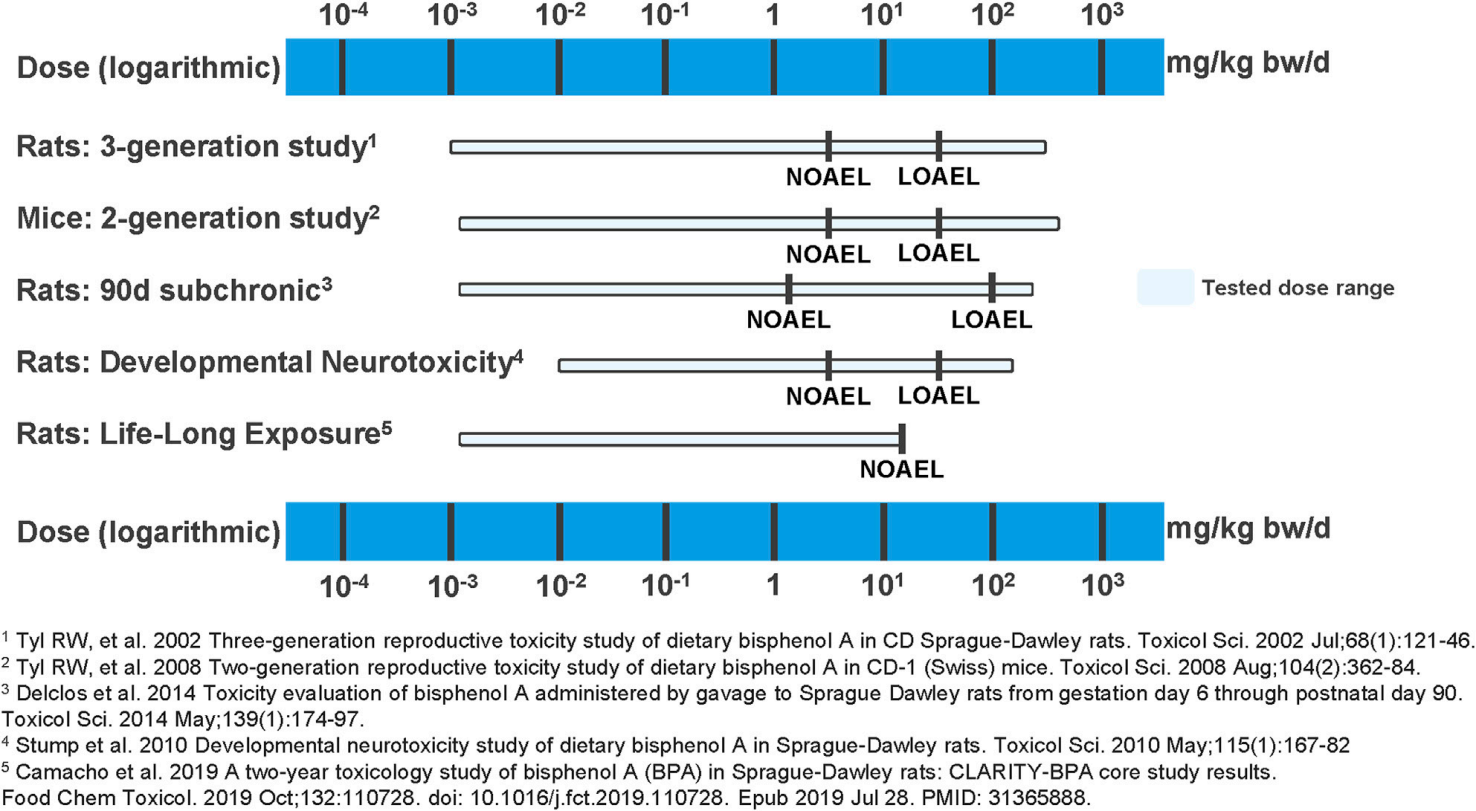
Overview of NOAELs and LOAELs derived from high quality BPA studies. In multigenerational studies in rats and mice (Tyl et al., 2002; Tyl et al., 2008), in a 90-day subchronic study in rats (Delclos et al., 2014), in a developmental neurotoxicity study in rats (Stump et al., 2010), and in a life-long exposure study in rats (Camacho et al., 2019), no BPA exposure-related adverse effects have been found at the tested lower dose end. Adverse and BPA treatment-related effects were only observed at doses ≥50,000 µg/kg-bw/day.

Finally, the NOAEL of 25,000 µg BPA/kg-bw/day proposed for the Core Study in the present evaluation aligns with other high-quality, long-term oral toxicity studies of BPA including multi-generational studies and a developmental neurotoxicity study. All of these studies consistently suggest that BPA does not pose a toxicological hazard below doses of 50,000 µg/kg-bw/day (Camacho et al., 2019; Delclos et al., 2014; Stump et al., 2010; Tyl et al., 2002; Tyl et al., 2008). The LOAELs from these studies range from 50,000 to 100,000 µg BPA/kg-bw/day (Figure 2).

## 5 Conclusion

Our evaluation of statistically significant findings from the CLARITY-BPA Core Study supports the conclusion that BPA does not produce adverse effects at doses up to 25,000 µg/kg-bw/day. While some non-neoplastic changes were observed in female reproductive organs and male pituitary glands at this highest dose, these findings lack consistency across timepoints, were not reproduced in parallel Grantee studies, and are consistent with normal aging processes in rats. Importantly, none of the effects demonstrated toxicological relevance or progression to neoplasia.

Despite concerns suggesting potential adverse effects at lower doses and proposing NMDRs, these interpretations are not supported by the Core Study authors or independent analyses, which found no evidence of NMDR effects in the Core Study. Furthermore, high-quality, long-term oral toxicity studies of BPA corroborate the absence of toxicological effects at doses below 50,000 µg BPA/kg-bw/day.

Therefore, the present evaluation reveals that a NOAEL of 25,000 µg BPA/kg-bw/day is reasonable and toxicologically justified for the CLARITY-BPA Core Study.

Financial support was provided by the Polycarbonate/BPA Global Group at the American Chemistry Council. The analysis and conclusions presented herein are those of the authors and not of their employer or the sponsor.

## Data Availability

The original contributions presented in the study are included in the article/supplementary material, further inquiries can be directed to the corresponding author.
